# Supplementary material for: A pharmacokinetic model of lead absorption and calcium competitive dynamics
Source: Sci Rep. 2019 Oct 2;9:14225. doi: 10.1038/s41598-019-50654-7 (PMC6775169; doi:10.1038/s41598-019-50654-7)
Supplement: Supplementary file 1 — Appendix A: Dependence on sigmoidal parameters [file 41598_2019_50654_MOESM1_ESM.pdf]

A pharmacokinetic model of lead absorption and calcium competitive dynamics

Anca Rădulescu*,[1], Steven Lundgren[2]

[1] Department of Mathematics, State University of New York at New Paltz; New York, USA; Phone: (845) 257-3532; Email: radulesa@newpaltz.edu;

[2] Department of Mechanical Engineering, State University of New York at New Paltz; New York, USA
*Corresponding author

Appendix A: Dependence on sigmoidal parameters

The model uses sigmoidal functions to capture two nonlinear phenomena: the saturable aspect of molecular transport between compartment pairs, and the gating mechanism preventing excessive calcium from being absorbed into the system at the digestive epithelial level. Sigmoidals shapes were chosen because they represent mathematically the saturation phenomenology: they increase from a left horizontal asymptote at zero towards a saturation horizontal asymptote value of 1, going through a high sensitivity window (where small changes in the input produce sizable effects in the response), surrounding an inflection point.

The position and width of the sensitivity window are modulated by the parameter pair b and θ (for the first function), and by a and τ (for the second function). Hence, for the first sigmoidal function, b represents the combined compartmental levels of lead and calcium for which the saturation mechanism of molecular transport to another compartment is most sensitive; θ represents the value of this maximal sensitivity. For the second sigmoidal, a represents the level of blood calcium at which the thresholding mechanism is the most sensitive, and τ measures this sensitivity.

In all our numerical experiments presented in the main text, these parameters were assigned the values $a = b = 0.6$, $\theta = 6$ and $\tau = 4$. These values were loosely tuned to lie within intervals that lead to levels of the system variables within the observed, empirical range. However, the precise values are not significant, since sizable variations of these parameter values on either side (larger or smaller) were shown to lead to qualitatively similar results (Figures 1–4 below). Of course, as one would expect, much larger perturbations of the sigmoidal parameters would eventually change the phenomenology of the system; based on our tuning to empirical ranges of the variables, we consider those parameter ranges unbiological.

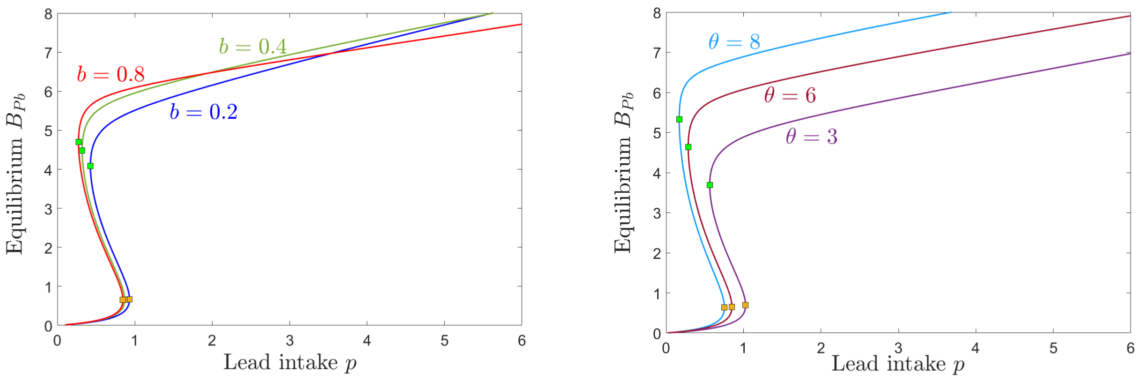

Figure 1: **Changes in the system response to increasing lead intake, as the sigmoidal parameters b and θ are varied. Left.** *Curves show the B_{Pb} projection of the equilibirum curve as p increases, for $\theta = 6$ and three values of b: $b = 0.2$ (blue), $b = 0.4$ (green) and $b = 0.8$ (red).* **Right.** *Curves show the B_{Pb} projection of the equilibirum curve as p increases, for $b = 0.6$ and three different values of θ: $\theta = 3$ (purple), $\theta = 6$ (brown) and $\theta = 8$ (cyan). All equilibirum curves exhibit a pair of saddle node bifurcation points, marked by green and yellow squares, respectively. The other parameters are fixed to $c = 10$, $k = 0.05$, $z = 1$, $s = 1$, $B = 0.8$, $a = 0.6$, $\tau = 4$.*

Figure 1a shows that the behavior of the steady states of the system in response to increasing lead intake only exhibits small changes in response to varying the sensitivity parameter b within the interval $[0.2, 0.8]$ (recall that the value used in the paper is within this interval: $b = 0.6$). Figure 1b illustrates how the behavior is affected by shifting the sensitivity window of the saturation mechanism by increasing θ in the interval $[3, 8]$ (the value used in the paper is $\theta = 6$). The behavior of the steady state in response to increasing p remains qualitatively the same, although the levels

of the various components of the equilibrium may chage slightly (e.g., the figure shows that the brain lead levels increase with increasing sensitivity θ within the biological range).

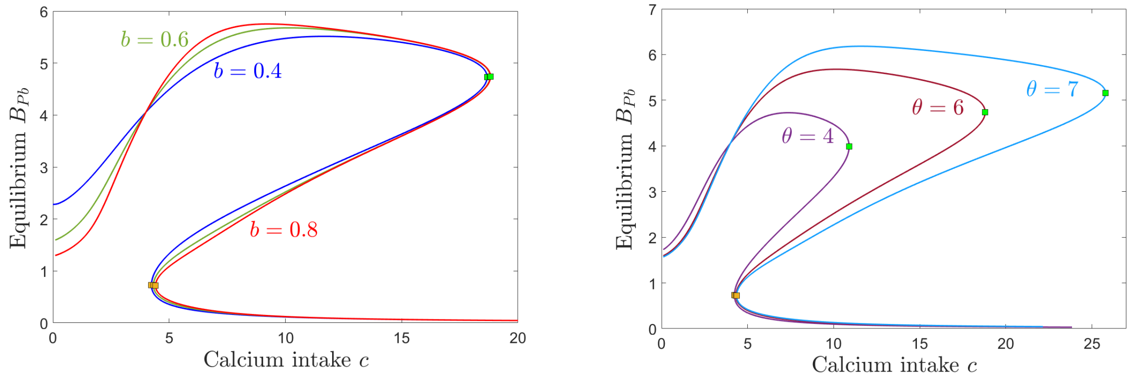

Figure 2: **Changes in the system response to increasing calcium intake, as the sigmoidal parameters b and θ are varied. Left.** *Curves show the B_{Pb} projection of the equilibirum curve as c increases, for $\theta = 6$ and three values of b: $b = 0.4$ (blue), $b = 0.6$ (green) and $b = 0.8$ (red).* **Right.** *Curves show the B_{Pb} projection of the equilibirum curve as p increases, for $b = 0.6$ and three different values of θ: $\theta = 4$ (purple), $\theta = 6$ (brown) and $\theta = 7$ (cyan). All equilibirum curves exhibit a pair of saddle node bifurcation points, marked by green and yellow squares, respectively. The other parameters are fixed to $p = 0.5$, $k = 0.05$, $z = 1$, $s = 1$, $B = 0.8$, $a = 0.6$, $\tau = 4$.*

Figure 2 shows that this remains the situation when one tracks the evolution of steady states in response to increasing the calcium intake c. The qualitative behavior of the equilibria is preserved for b and θ varied within the specified intervals around $b = 0.6$, $\theta = 6$. Since the presence of lead in the brain is the focus of this paper, we chose to illustrate the corresponding component of the equilibrium curve, showing that its evolution with c changes very little when b is varied, and slightly shifts up when θ is increased.

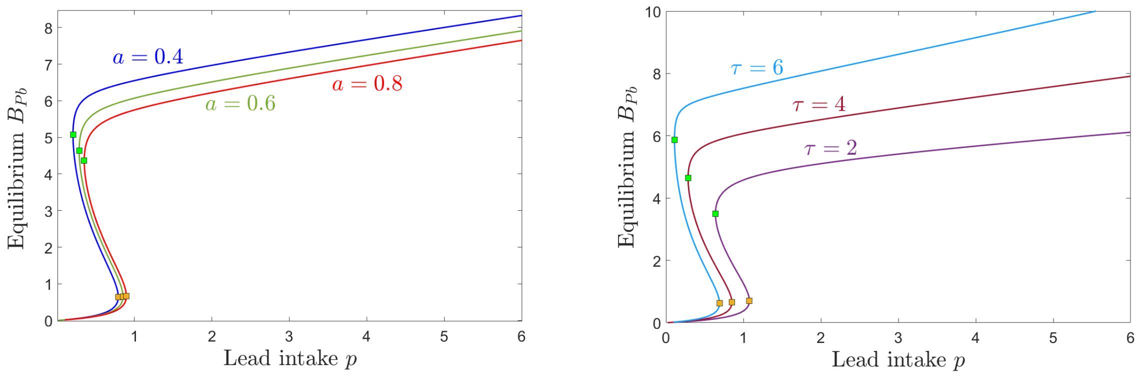

Figure 3: **Changes in the system response to increasing lead intake, as the sigmoidal parameters a and τ are varied. Left.** *Curves show the B_{Pb} projection of the equilibirum curve as p increases, for $\tau = 4$ and three values of a: $a = 0.4$ (blue), $a = 0.6$ (green) and $a = 0.8$ (red).* **Right.** *Curves show the B_{Pb} projection of the equilibirum curve as p increases, for $a = 0.6$ and three different values of τ: $\tau = 2$ (purple), $\tau = 4$ (brown) and $\theta = 6$ (cyan). All equilibirum curves exhibit a pair of saddle node bifurcation points, marked by green and yellow squares, respectively. The other parameters are fixed to $c = 10$, $k = 0.05$, $z = 1$, $s = 1$, $B = 0.8$, $b = 0.6$, $\theta = 6$.*

Figures 3 and 4 illustrate a similar sensitivity analysis around changes in a and τ. The system's response to increases in both lead and calcium intakes remains qualitatively the same, when varying a and τ within the specified biological intervals, with a quantitative effect of overall increasing brain lead if the threshold value τ is increased, or if the sensitivity a is decreased.

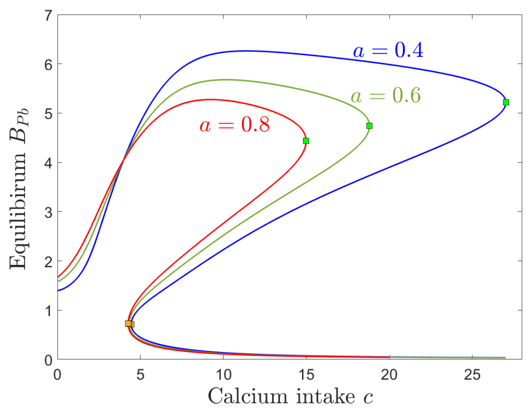

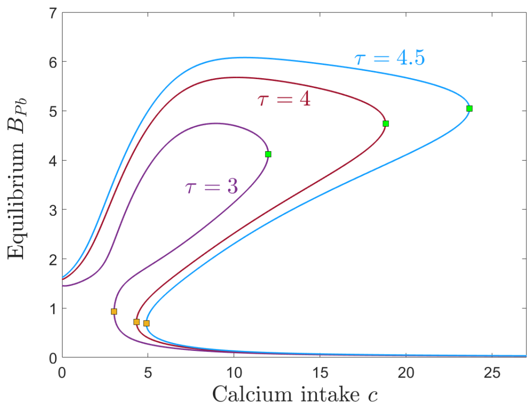

Figure 4: **Changes in the system response to increasing calcium intake, as the sigmoidal parameters a and τ are varied. Left.** *Curves show the B_{Pb} projection of the equilibirum curve as c increases, for $\tau = 4$ and three values of a: $a = 0.4$ (blue), $a = 0.6$ (green) and $a = 0.8$ (red).* **Right.** *Curves show the B_{Pb} projection of the equilibirum curve as p increases, for $a = 0.6$ and three different values of τ: $\tau = 3$ (purple), $\tau = 4$ (brown) and $\tau = 4.5$ (cyan). All equilibirum curves exhibit a pair of saddle node bifurcation points, marked by green and yellow squares, respectively. The other parameters are fixed to $p = 0.5$, $k = 0.05$, $z = 1$, $s = 1$, $B = 0.8$, $b = 0.6$, $\theta = 6$.*
